# Identifying depression disorder using multi-view high-order brain function network derived from electroencephalography signal

**DOI:** 10.3389/fncom.2022.1046310

**Published:** 2022-10-27

**Authors:** Feng Zhao, Tianyu Gao, Zhi Cao, Xiaobo Chen, Yanyan Mao, Ning Mao, Yande Ren

**Affiliations:** ^1^School of Computer Science and Technology, Shandong Technology and Business University, Yantai, China; ^2^College of Oceanography and Space Informatics, China University of Petroleum (Huadong), Qingdao, Shandong, China; ^3^Department of Radiology, Yantai Yuhuangding Hospital, Yantai, Shandong, China; ^4^Department of Radiology, The Affiliated Hospital of Qingdao University, Qingdao, China

**Keywords:** EEG, major depression disorder, brain function networks, matrix variate normal distribution, high-order brain function networks

## Abstract

Brain function networks (BFN) are widely used in the diagnosis of electroencephalography (EEG)-based major depressive disorder (MDD). Typically, a BFN is constructed by calculating the functional connectivity (FC) between each pair of channels. However, it ignores high-order relationships (e.g., relationships among multiple channels), making it a low-order network. To address this issue, a novel classification framework, based on matrix variate normal distribution (MVND), is proposed in this study. The framework can simultaneously generate high-and low-order BFN and has a distinct mathematical interpretation. Specifically, the entire time series is first divided into multiple epochs. For each epoch, a BFN is constructed by calculating the phase lag index (PLI) between different EEG channels. The BFNs are then used as samples, maximizing the likelihood of MVND to simultaneously estimate its low-order BFN (Lo-BFN) and high-order BFN (Ho-BFN). In addition, to solve the problem of the excessively high dimensionality of Ho-BFN, Kronecker product decomposition is used for dimensionality reduction while retaining the original high-order information. The experimental results verified the effectiveness of Ho-BFN for MDD diagnosis in 24 patients and 24 normal controls. We further investigated the selected discriminative Lo-BFN and Ho-BFN features and revealed that those extracted from different networks can provide complementary information, which is beneficial for MDD diagnosis.

## Introduction

Major depressive disorder (MDD) is a common mental illness. A recent report by the World Health Organization (WHO) showed that approximately 340 million people worldwide suffer from depression of different severities (Gray et al., 2020; Liu et al., 2020). During a depressive episode, patients experience physical symptoms such as insomnia, poor diet, and psychological symptoms. They gradually lose interest in things, which often leads to suicide in severe cases (Kraus et al., 2019; Orsolini et al., 2020). In recent years, EEG has been widely used in clinical research (Liu et al., 2008; Wang et al., 2013) to provide support for the diagnosis of MDD. Owing to its high temporal resolution, which can detect short-term changes in neural signals, EEG is very suitable for capturing rapid and dynamic changes in the brain (Teplan, 2002; Fingelkurts et al., 2007; Loo et al., 2016).

The existing analysis methods based on EEG data can be classified into two categories. The first category is based on the independent characteristics of each EEG channel (Bachmann et al., 2018; Lotte et al., 2018; Newson and Thiagarajan, 2019). For example, Hosseinifard et al. (2013) extracted four EEG band powers and four non-linear features to classify 45 depressed patients and 45 normal subjects, achieving 90% accuracy. Boonyakitanont et al. (2020) analyzed the classification results of epilepsy, based on classic linear and non-linear features in EEG, and explored the most discriminative features according to their experimental results. Although these methods can provide useful information for exploring the relationship between depression and EEG signals, they only extract the isolated features of each channel and ignore the correlation among different channels. In fact, experiments have shown that even for the simplest task, the brain needs to coordinate multiple regions. Therefore, the independent analysis of channel characteristics without considering their connections cannot comprehensively capture useful discriminative information in the brain (Abrams et al., 2013; Vecchio et al., 2013; Chu et al., 2015).

The second category corresponds to methods based on the brain function network (BFN), which can capture the relationship between channels (Dell’Acqua et al., 2021; Yasin et al., 2021). For example, Zhang et al. (2020) extracted discriminative features from a constructed phase lag index (PLI) matrix using graph theory, classified 24 depression patients and 29 normal controls, and finally achieved an accuracy of 93.31%. Li et al. (2017) compared the coherence relationship between MDD and normal control (NC). Their experiment revealed that the connections capable of discriminating between MDD and NC were mainly distributed in the left hemisphere of the brain, particularly in the parietal and temporal regions. To date, many popular connectivity indicators have been proposed, including the phase-locking value (PLV), phase slope index (PSI), PLI, and weighted phase-lag index (WPLI). These methods capture features from different perspectives and have achieved few results in patients’ classification (MohanBabu et al., 2021; Cao et al., 2022).

These studies have demonstrated the effectiveness of the functional connectivity in the study of depression. However, traditional functional connectivity, which we refer to as low-order BFN (Lo-BFN), only reflects the pairwise relationship between EEG channels while ignoring the relationship among multiple channels. As mentioned above, even for the simplest task, the brain needs the coordination of multiple brain regions; thus, analyzing only the relationship between two channels is not sufficient to explore the relationship among multi-EEG channels. In fact, in a biological sense, multiple distinct channels that are structurally separated may also be functionally tightly coordinated. We refer to a network that can reflect multiple EEG channels as high-order BFN (Ho-BFNs). To the best of our knowledge, few studies have used EEG-based Ho-BFNs for MDD diagnoses. Therefore, in our present study, we aim at exploring: (1) how to construct Ho-BFN, capable of reflecting the connection relationship among multiple channels, in order to further assist in exploring the deep-level connection relationship of the brain; and (2) to what extent integrating Ho-BFN and Lo-BFN can improve the accuracy of MDD diagnosis.

**FIGURE 1:**
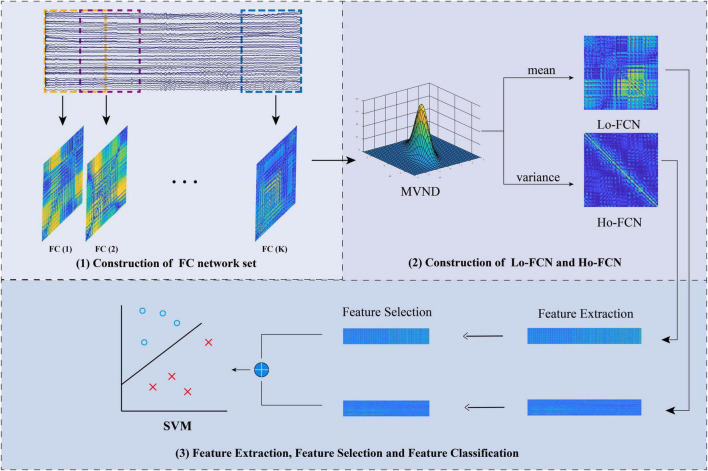
The flowchart of the proposed framework.

Based on the above analysis, we propose a framework, which can simultaneously capture both the Lo-BFN and Ho-BFN from EEG signals for MDD diagnosis. Figure 1 shows the workflow of the proposed framework. First, we used the sliding window strategy to divide the entire time series into multiple epochs and construct the BFN for each epoch [Figure 1 (1)]. Then, the constructed BFN are used as samples to simultaneously estimate the low- and high-order networks by maximizing the likelihood of the matrix variate normal distribution (MVND) [Figure 1 (2)]. In particular, the mean matrix of the Gaussian distribution, which expresses the Lo-BFN reflects the mean value of the dynamic connection relationship between every pair of channels. The covariance matrix, which expresses the Ho-BFN, reflects the pairwise interaction pattern between functional connectivity (FC) involving multiple channels. In addition, considering the high dimension of the generated covariance matrix, we use the Kronecker product decomposition to reduce its dimensions. Finally, the low-and high-order BFN are fused at the feature layer to predict the target class label (MDD or NC) for a given testing subject.

In fact, similar high-order network methods have been applied in the field of functional magnetic resonance imaging (fMRI) (Zhao et al., 2018, 2021; Zhou et al., 2018), where experimental results have shown that the high-order network method can explore more advanced features on the basis of the traditional low-order network. However, owing to the low temporal resolution of fMRI, the Gaussian distribution leads to inaccurate estimation results. In contrast, EEG has the characteristic of high temporal resolution, which can provide more samples for the calculation of the Gaussian distribution and more accurately estimate the Gaussian distribution of the BFNs. To the best of our knowledge, EEG-based high-order networks have not yet been used for MDD diagnosis. Thus, applying a high-order network to EEG-based MDD diagnosis is clinically and scientifically valuable.

Overall, this study makes the following contributions: (1) a high-order network method is proposed to first express the deeper connection relationships among multiple channels and reflect the deep connection mechanism of the brain, which has not been explored previously in the literature. (2) Based on the MVND estimation, we propose a framework that constructs and fuses both low-and high-order networks for MDD classification. The experimental results demonstrate that the proposed framework can distinguish between MDD and NC better than traditional methods.

## Materials and data preprocessing

In this study, we used a public depression dataset from MODMA (Cai et al., 2020). The total number of samples was 48, including 24 patients with MDD (12 males and 12 females) and 24 normal controls (15 males and 9 females). All subjects gave written informed consent before the start of the experiment. Detailed demographic information of these subjects is summarized in Table 1. All participants were right-handed, and their education level was primary school or above. To ensure that the mental state of the subjects was not affected by other factors, for the normal control group, we excluded those with personal and family mental history, and also investigated whether the subjects had alcohol dependence or drug use in the past year. For MDD patients, we tested the degree of depression using the PHQ-9 standard (Spitzer et al., 1999) to ensure that the PHQ-9 score was greater than or equal to five. In addition, none of the patients with depression received psychotherapy within 2 weeks.

**TABLE 1:** Demographic information of the subjects.

	MDD	NC	*P*-values
Gender (M/F)	12/12	15/9	0.2059^a^
Age (mean ± SD)	30.9 ± 21.1	30.9 ± 20.1	0.9880^b^
PHQ-9 (mean ± SD)	18.3 ± 7.3	2.6 ± 2.6	0.0000^b^
GAD-7 (mean ± SD)	13.4 ± 11.4	2.1 ± 4.9	0.0000^b^

MDD, major depression disorder; NC, normal control; M, male; F, female; PHQ-9, Patient Health Questionnaire-9 item; GAD-7 (Spitzer et al., 2006), Generalized Anxiety Disorder-7. ^a^The *p*-value obtained by chi-square test; ^b^The *p*-value obtained by two-sample *t*-test.

A 128 channel HydroCel Geodesic Sensor Net (HCGSN) with a Cz reference is used for data acquisition. During the collection process, to reduce the interference of EEG data, the subjects sat in a chair in a dark room and were asked to close their eyes and avoid moving.

For each subject, we conduct 0.1–40 Hz filtering and 48–52 Hz depression filtering to remove the baseline drift and electrical interferences from the data. The REST (Yao, 2001) method is then used to re-reference the data, which is a method of standardizing scalp EEG recording to the infinity point, helpful to more accurately restore the data of the frontal region. Finally, the ASR method was used to remove bad epochs (produced by eye blinks, muscle activity, sensor motion, etc.) from the EEG data. At the same time, we also removed the beginning part and the end part of the whole data to ensure that the subjects were in a stable state. In the end, we get the percentage of clean data is 89%. In this study, the frequency bands of interest are delta (1–4 Hz) and theta (4–8 Hz), computed by fast Fourier transform (FFT). The aforementioned processing steps were performed in MATLAB R2018b.

## Methods

In this section, we introduce the construction of Lo-BFNs and Ho-BFNs using the MVND method. First, we demonstrate how to construct a set of BFNs using a sliding window. We then introduce MVND to simultaneously construct Lo-BFN and Ho-BFN. Finally, we elaborate on the framework for feature extraction, fusion, and classification.

### Construction of phase lag index correlation matrices

Let *x*_*i*_ = (*x*_*i*1_, *x*_*i*2_,⋯,*x*_*iC*_)(*i* = 1, 2,⋯,*N*) denote the EEG time series associated with the *i*-th channel, where *C* is the number of time-sample points and *N* represents the number of subjects.

We adopted the sliding window method to generate the sequence of BFNs, which can reflect the dynamic changes in the correlation between channels. Suppose that the window width and step size are *W* and *S*, respectively. We can then generate *K* windows for a given EEG time series, where *K* = [(*C* − *W*)/*S*] + 1.

For each sliding window, we adopt the PLI method to construct the corresponding BFNs and obtain a series of BFNs {ℋ^(1)^, ℋ^(2)^,⋯,ℋ^(*k*)^} (*k* = 1, 2,⋯,*K*). The PLI method is widely used in EEG classification tasks because it can exclude the influence of the common source problem and estimate the degree of phase synchronization more accurately. For two EEG signals *i* and *j*, the PLI was computed as follows:


(1) $$P\,L\,I_{i\,j}=\left|\frac{1}{n}\,\sum_{n=1}^{N}s\,i\,g\,n\,\left(\Delta \,\phi_{i}\,\left(t_{n}\right)-\Delta \,\phi_{j}\,\left(t_{n}\right)\right)\right|,$$

where Δϕ_*i*_(*t*) and Δϕ_*j*_(*t*) are the phase values of EEG signal *i* and *j* at time *t*, respectively, *sign* is the sign function, and *t*_*n*_ denotes the range of the entire time series. Therefore, we generate a 128 × 128 matrix as BFN under each sliding window.

### Construction of low and high order brain function networks based on matrix variate normal distribution

**FIGURE 2:**
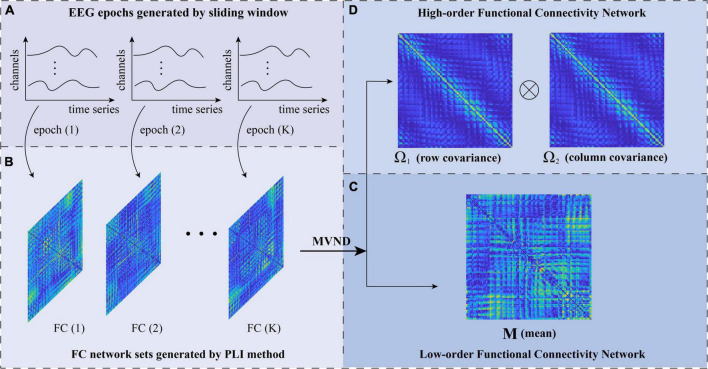
The flowchart of low- and high-order brain function networks (BFN) construction based on matrix variate normal distribution (MVND). **(A)** EEG epochs generated by sliding window. **(B)** FC network sets generated by PLI method. **(C)** Low-order functional connectivity network. **(D)** High-order functional connectivity network.

After we obtained a set of BFNs by applying the PLI method to each sliding window (Figures 2A,B), we adopted the MVND (Gupta and Nagar, 2018) method to simultaneously construct the low-and high-order BFNs. Suppose the correlation between the EEG signal *x* and *y* is a random variable *h*_*xy*_ with a normal distribution. Then, the sequence of BFNs ℋ = (*h_xy_*)_*N*×*N*_ follows the multivariate normal distribution, which is defined as follows:


(2) ℋ∼N⁢(M,Σ),

where M ∈ *R^N^*^×^*^N^* is the population mean or mathematical expectation of ℋ. Σ is the population covariance matrix of ℋ. It should be noted that, M represents the average BFN under different windows, which is still a low-order correlation (Figure 2C). The covariance matrix Σ represents the correlation between random variables *h*. Therefore, Σ reflects information from multiple channels, which is a Ho-BFN.

Considering that the dimension of the covariance matrix Σ with a size of 128^2^×128^2^ is too high, the Kronecker product decomposition, i.e., Σ = Ω_1_ ⊗ Ω_2_, is used to reduce the dimension. Therefore, ℋ can be expressed as:


(3) $$ℋ∼N\,\left(\text{M},{\text{Ω}}_{1}⊗\Omega_{2}\right),$$

where Ω_1_, Ω_2_ ∈ *R^N^*^×^*^N^* represent the row covariance and column covariance (Figure 2D) of the original matrix ℋ, respectively. Because the correlation of PLI adopted in this study is undirected, the constructed correlation matrix is symmetric, so the row covariance Ω_1_ and column covariance Ω_2_ are equal. Specifically, we used the maximum likelihood estimation (MLE) method to solve the mean and variance of MVND (Zhang and Schneider, 2010; Gupta and Nagar, 2018), the formula of which is as follows:


(4) $$M=\frac{1}{K}\,\sum_{k=1}^{K}{ℋ}^{\left(k\right)}.$$

Ω is obtained by iteration, and its initial condition is Ω = *I*, where *I* is the identity matrix. The formula is as follows:


(5) $$\Omega =\frac{1}{K\,N}\,\sum_{k=1}^{K}\left({ℋ}^{\left(k\right)}-M\right)\,\Omega^{-1}\,{\left({ℋ}^{\left(k\right)}-M\right)}^{T}.$$

### Feature extraction, selection, and classification

In this section, we introduce how to extract, select, and classify features. For each subject, we used the MVND method to obtain the Lo-BFN and Ho-BFN and then vectorized the two matrices as the characteristics of the subject. Because the constructed matrix is symmetric, we retain only the effective features in the vectorization. Specifically, for an *n* × *n* BFN, the size after vectorization is (*n* × n–n)/2, where *n* is the number of EEG channels. Specifically, we use the linear fusion method to fuse the features of the low-order and high-order networks.

Considering the feature dimension too high, which may cause overfitting, we used the *t*-test and least absolute shrinkage and selection operator (LASSO) to extract the discriminative features. For this binary classification problem, both methods are classic and effective. We conducted a *t*-test on the positive and negative examples of the training data to extract the discriminative features, and then used LASSO to further remove redundant features. Suppose ω_*i*_ = (ω_*i*1_, ω_*i*2_,⋯,ω_*ir*_) is the weight of the eigenvector, where r is the number of features obtained from the *t*-test. Let *y* = {*y*_1_, *y*_2_,⋯,*y*_*d*_,} represent the feature set extracted from BFN and *I* the label of the corresponding BFN. For the positive example, the label is 1, whereas for the negative ones, the label is −1. The LASSO is calculated as:


(6) $$\frac{1}{2}\,\sum_{l=1}^{L}{||I^{l}-\left\langle \hat{y},\omega_{i}\right\rangle ||}_{2}^{2}+\lambda \,{||\omega_{i}||}_{1},$$

where λ is the parameter that controls the regular term *L*1−*norm*. Sparse feature selection can be achieved by setting a specific value for λ. Furthermore, we used an SVM classifier to classify the extracted features and identify the EEG signals of patients with depression.

## Results

The proposed method was evaluated by testing it for the classification of MDD and NC subjects. Additionally, we analyzed the feature weights of the Lo-BFN and Ho-BFN to identify the most discriminative EEG channels and brain regions for classifying MDD and NC.

For the problem of small sample dataset, a six-fold cross-validation (CV) method was adopted to evaluate the performance of the proposed method. This method can separate the training set and the test set, which can effectively avoid the problem of overfitting. At the same time, in order to avoid the chance of the experimental results, we repeated the process of cross-validation ten times, and finally took the average of the ten results as the final result. In the choice of the classifier, we chose the SVM classifier. The reason is that the essence of SVM is a convex optimization problem, which makes it unique in dealing with small sample data. There are three hyper-parameters in the training process: the coefficient λ of the regularization term in the lasso model, parameter *p* of the *t*-test, and penalty coefficient c of the SVM classifier. In our experiment, we tuned these parameters in the following ranges: λ ∈ [0.1 :0.1 :0.7], *p* ∈ [0.01 :0.01 :0.1], *c* ∈ [0.1 :0.1 :0.9]. Specifically, we nested three layers of loops, corresponding to three parameters, respectively. Under this condition, we fixed three different parameters before performing cross-validation.

### The influence of parameters on brain function networks

**FIGURE 3:**
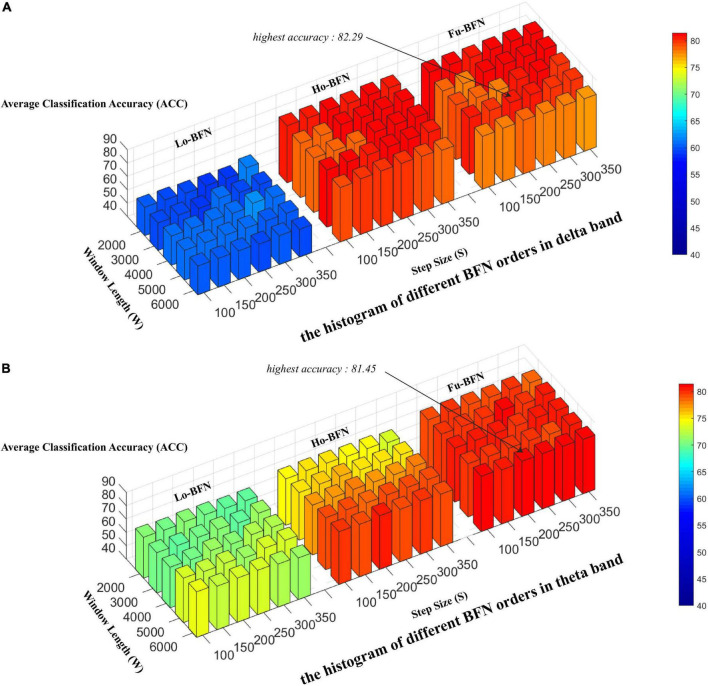
Effect of different sliding window parameters on classification accuracy. **(A)** The histogram of different brain function networks (BFN) orders in delta band. **(B)** The histogram of different BFN orders in theta band.

To estimate the normal distribution of the matrix, we used the sliding window method to generate BFN sequences as samples. Two key parameters of the sliding window affect the final classification results: the window width (W) and the step size (S). A small window can capture the short-term fluctuation of the signal more accurately, whereas a large window can estimate the BFN more stably. Therefore, we designed experiments to evaluate the classification accuracy under different window widths and steps. Specifically, the window width was set to [2,000, 3,000, 4,000, 5,000, and 6,000] and the step size was set to [100, 150, 200, 250, 300, and 350]. Figure 3 shows the experimental results. The three regions from left to right in the figure are Lo-BFN, Ho-BFN and the fusion of them (Fu-BFN). The y-axis represents the classification accuracy.

### Fusion results of the low-order brain function networks, high-order brain function networks, and fusion brain function networks

**FIGURE 4:**
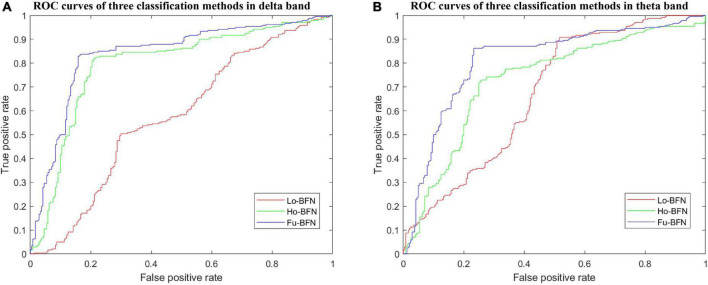
ROC curves of brain function networks (BFNs) in different bands. **(A)** ROC curves of three classification methods in delta band. **(B)** ROC curves of three classification methods in theta band.

We used the optimal parameters discussed in the previous section to build a classifier that was evaluated for MDD diagnosis. Similar to previous studies, we used six metrics to evaluate the classification performance: accuracy (ACC), sensitivity or true positive rate (TPR), specificity or true negative rate (TNR), precision or positive predictive value (PPV), negative predictive value (NPV), and F1 score. Higher values of these metrics indicate better classification performance. Table 2 reports the best results of MDD diagnosis with the Lo-BFN, Ho-BFN, and Fu-BFN strategies under the optimal parameters in the delta and theta bands. The best results are highlighted in bold font. In addition, we depict the ROC curves of the above classification results in Figure 4.

**TABLE 2:** Performance of different order brain function networks (BFNs) in different bands.

Frequency band	Network	ACC	TPR	TNR	PPV	NPV	F1
Delta	Lo-BFN	63.75 ± 0.04	60.69 ± 0.15	61.60 ± 0.37	65.96 ± 0.12	59.69 ± 0.07	65.03 ± 0.12
	Ho-BFN	80.33 ± 0.20	79.31 ± 0.19	80.00 ± 0.26	82.14 ± 0.05	76.92 ± 0.32	80.70 ± 0.05
	Fu-BFN	**83.54 ± 0.06**	**84.14 ± 0.42**	**82.40 ± 0.10**	**84.72 ± 0.11**	**81.75 ± 0.08**	**84.43 ± 0.09**
Theta	Lo-BFN	74.17 ± 0.29	73.58 ± 0.18	64.17 ± 0.08	73.61 ± 0.25	77.92 ± 0.33	76.17 ± 0.40
	Ho-BFN	79.21 ± 0.09	75.33 ± 0.05	79.17 ± 0.20	77.50 ± 0.12	78.61 ± 0.17	78.56 ± 0.55
	Fu-BFN	**81.46 ± 0.24**	**84.17 ± 0.23**	**80.25 ± 0.11**	**79.81 ± 0.06**	**81.94 ± 0.34**	**78.92 ± 0.26**

Reports the best results of MDD diagnosis with the Lo-BFN, Ho-BFN, and Fu-BFN strategies under the optimal parameters in the delta and theta bands. The best results are highlighted in bold font.

### The most discriminative features for major depressive disorder diagnosis

To explore why the Ho-BFN improves MDD classification performance from a physiological perspective, we identified a set of the most discriminative features. Specifically, we computed the frequency at which features were selected in the Lo-BFN and Ho-BFN in the cross-validation of the two bands. A higher frequency indicated that the corresponding feature was more discriminative.

**FIGURE 5:**
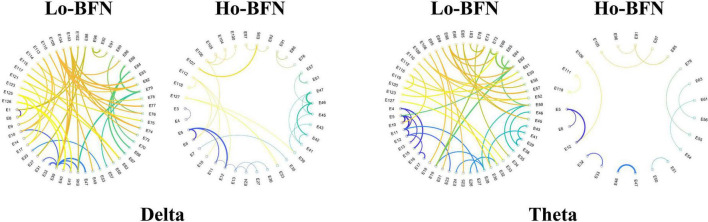
The discriminative functional connectivity (FC) of low- and high-order brain function networks (BFNs) in delta and theta band. The thicker the line, the stronger the discrimination.

**FIGURE 6:**
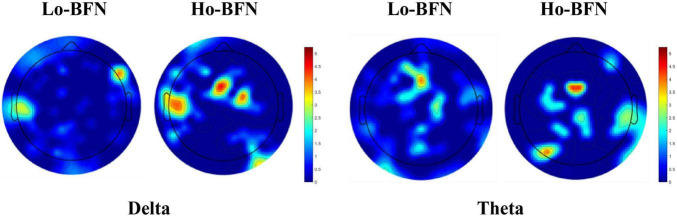
The discriminative channels on topographic map. The warmer the color, the stronger the discrimination of the corresponding channel.

As shown in Figure 5, we visualized the most discriminative features using circular graphs, where the nodes represent the EEG channels, the line represents the FC relationship between the two channels, and the line thickness indicates the discriminative ability. Because not all FCs have good discrimination in disease classification, we select the top 50 discriminative connections and display them. To clearly express the physiological location of the connection and the discriminative characteristics of the Lo-BFN and Ho-BFN, we counted the channels involved in the discriminative FC and plotted them into a topographic map, which is shown in Figure 6.

## Discussion

This study provides a mean for assisting with the assessment of clinical suicidal ideation. We analyzed EEG signals from a new perspective, taking into account the connections between multiple channels, and conducted several experiments. We provide a more detailed discussion of the results of these experiments.

Figure 3 shows the accuracy of Lo-BFN and Ho-BFN with different parameters. We derive the following conclusions: (1) the accuracy of the classification is sensitive to two parameters, i.e., window width and step size. Specifically, for Lo-BFN, the highest accuracy was achieved when *W* = 4 and *L* = 3. For Ho-BFN and their fusion, the highest accuracy was achieved when *W* = 5 and *L* = 4. (2) When using the same sliding window parameters, the results of Ho-BFN and Fu-BFN significantly outperformed those of Lo-BFN. (3), Fu-BFN had a higher discrimination ability in the delta band compared to the theta band.

To further analyze the reliability of the proposed method, we selected the optimal parameters for the analysis. From the experimental results in Table 2 and Figure 4, it can be seen that (1) the classification accuracy of the Ho-BFN is significantly higher than that of the traditional Lo-BFN. (2) Fu-BFN can further improve the classification accuracy, which indicates that Lo-BFN and Ho-BFN can be complementary to enhance classification performance. (3) The Lo-BFN accuracy of the theta band was higher than that of the delta band, whereas in the proposed Ho-BFN, the accuracy in the delta band was higher than that in the theta band.

We further identified important brain regions associated with MDD, based on the high frequency connections involved in our method. The results in Figures 5, 6 reveal the following findings: (1) some high-order and low-order discriminative features are shared, and some are different. Ho-BFN can generate more discriminative features than Lo-BFN. This dissimilar feature between Lo-BFN and Ho-BFN explains the improved classification accuracy. (2) Lo-BFN and Ho-BFN are significantly different in the frontal region and left temporal region of the electrodes in the delta band. The frontal region is central to the emotions and thought processes that translate into personality (Lee et al., 2020). Therefore, differences in the frontal region may be an important cause of memory loss and inattention in patients with MDD. The function of the temporal lobe is centered on memory and emotion (AlShorman et al., 2020), which corresponds to the clinical manifestations of MDD, such as emotional instability. Zhang et al. (2020) found that significant alterations of brain synchronization occurred in frontal, temporal, parietal-occipital regions of left brain and temporal region of right brain in MDD patients. Another studies (Li et al., 2017; Kiriyama et al., 2020) found that the left temporal region plays a critical role in MDD patients. Combined with our findings, the frontal and left temporal lobes may play an important role in the pathogenesis of MDD. In addition, we found a difference in the left parietal lobe in the theta band. The left parietal lobe is located behind the frontal lobe, which is the starting point of sensory processing within the brain. Some changes in the parietal lobe may reflect the cognitive problems of MDD patients, which is similar to findings in the literature (Bobde et al., 2018; Li et al., 2021).

Although the Ho-BFN proposed in this study can be helpful in the diagnosis of patients with MDD, several limitations could be discussed. As in many previous studies (Shao et al., 2021; Zhu et al., 2021), the first limitation relates to the small sample size. In the future, further validation of the reliability of our method with larger sample sizes (e.g., using other publicly available datasets) should be considered to avoid possible overfitting issues. Therefore, caution must be exercised when applying this method in a clinical setting. Second, the Ho-BFN is constructed using MVND, which analyzes the discrete degree of the time series of correlation changes as a whole, but does not consider short-term changes in the series. Finally, we compare our accuracy with the highest accuracy in the field, and find that our accuracy is still has room to improve. The next step is to consider combining our features with theirs to further improve the performance of identifying MDD.

## Conclusion

In this study, we proposed a method for EEG-based MDD diagnosis. We constructed the Lo-BFN and Ho-BFN, which can capture high-order relationships across different EEG channels. This method treats the time-varying sequence of the correlation matrix between each pair of channels as a normal distribution and simultaneously estimates the Lo-BFN and Ho-BFN. The experimental results have shown that: (1) Compared to traditional Lo-BFN, Ho-BFN can further extract discriminative features and improve classification accuracy. Moreover, the features of the Lo-BFN and Ho-BFN can complement each other in MDD classification. Their fusion can further improve the diagnosis performance. (2) We found that the most discriminative brain regions were mainly located in the left frontal, right frontal, and left temporal regions, which is consistent with previous studies.

## Data availability statement

Publicly available datasets were analyzed in this study. This data can be found here: http://modma.lzu.edu.cn/data/index/.

## Ethics statement

The studies involving human participants were reviewed and approved by the Local Ethics Committee for Biomedical Research at the Lanzhou University Second Hospital in accordance to the Code of Ethics of the World Medical Association (Declaration of Helsinki). Written informed consent to participate in this study was provided by the participants’ legal guardian/next of kin.

## Author contributions

FZ: conceptualization, methodology, and writing—review and editing. TG: conceptualization, software, writing—original draft, methodology, formal analysis, investigation, and validation. ZC: validation. XC, YM, NM, and YR: writing—review and editing. All authors contributed to the article and approved the submitted version.
